# Gene expression variation in the brains of harvester ant foragers is associated with collective behavior

**DOI:** 10.1038/s42003-020-0813-8

**Published:** 2020-03-05

**Authors:** Daniel Ari Friedman, Ryan Alexander York, Austin Travis Hilliard, Deborah M. Gordon

**Affiliations:** Stanford University, Department of Biology, Stanford, CA 94305 USA

**Keywords:** Gene expression, Ecological genetics, Molecular evolution

## Abstract

Natural selection on collective behavior acts on variation among colonies in behavior that is associated with reproductive success. In the red harvester ant (*Pogonomyrmex barbatus*), variation among colonies in the collective regulation of foraging in response to humidity is associated with colony reproductive success. We used RNA-seq to examine gene expression in the brains of foragers in a natural setting. We find that colonies differ in the expression of neurophysiologically-relevant genes in forager brains, and a fraction of these gene expression differences are associated with two colony traits: sensitivity of foraging activity to humidity, and forager brain dopamine to serotonin ratio. Loci that were correlated with colony behavioral differences were enriched in neurotransmitter receptor signaling & metabolic functions, tended to be more central to coexpression networks, and are evolving under higher protein-coding sequence constraint. Natural selection may shape colony foraging behavior through variation in gene expression.

## Abstract (web summary)

Friedman et al. examine gene expression in the brains of red harvester ant foragers. They show that colonies differ from each other in the expression of neurophysiologically-relevant genes, and that natural selection may shape colony foraging behavior by acting on this variation among colonies in gene expression.

## Introduction

The collective behavior of a social insect colony arises from the responses of individuals to interactions with others and with the environment^1,2^. Behavioral differences among colonies drive ecologically important variation in reproductive success^3–5^, and thus evolution in populations of colonies^6–8^. Colonies are reproductive individuals, so selection acts on colony behavior. Genomic variation among colonies, the raw material for selection^9^, may be reflected in differences among colonies in worker gene expression and neurophysiology^5,10–14^.

The biogenic amine neurotransmitters such as dopamine and serotonin influence ant worker behavior through their role in modulating sensory sensitivity to stimuli^15–18^. Dopaminergic neural circuitry is important in the regulation of foraging activity in ants and other insects^19–21^, as well as in the integration of water- and thirst-related stimuli^22,23^. In ants, biogenic amine neurophysiology has primarily been studied in controlled indoor conditions, to investigate why groups of nestmates differ in task performance or reproductive status^13,21,24–28^. Here, we extend these studies to consider how differences among colonies in forager neurophysiology are associated with variation in colony behavior in natural conditions.

Colonies of the red harvester ant (*Pogonomyrmex barbatus*) forage for seeds in the desert of the Southwest USA where water stress is an important ecological constraint^9^. Foraging requires a tradeoff between the gain of nutrition and water from seeds, and the costs of water loss by foragers exposed to desiccating conditions^29^. This tradeoff is regulated by olfactory interactions inside the nest between outgoing and returning foragers^30,31^. The rate of interaction with returning foragers provides outgoing foragers with feedback on food availability^32^, which they integrate with their recent experience of outside conditions^33,34^. Colonies of *P*. *barbatus* differ in the regulation of foraging in response to dry conditions, and these differences persist over years in successive cohorts of foragers within a colony^9,35,36^. As with other social insect species, neurophysiological differences among *P. barbatus* foragers associated with biogenic amine neurotransmitter signaling pathways^28^ may influence their sensitivity to interactions, and thus how the colony regulates foraging activity as conditions change. Manipulative experiments in the field show that hydrated foragers go on significantly more foraging trips than unhydrated nestmates, and that an increase in forager brain dopamine level also increases foraging activity^28,37^. The reproductive success of a *P. barbatus* colony, in offspring colonies, is associated with how the colony regulates foraging in dry conditions, and colonies resemble their mothers in how they regulate foraging activity in dry conditions^9^. This indicates that natural selection is acting on the collective regulation of foraging activity in *P. barbatus*^2^.

Here, we ask how gene expression in individual forager brains is associated with phenotypic variation across colonies in a natural setting. To characterize behavioral and physiological variation, we collected actively foraging ants from nine *P. barbatus* colonies that varied in two traits: (1) sensitivity of foraging activity to humidity, measured as the decrease in foraging trips made by the colony per percentage reduction in humidity, and (2) average forager brain dopamine-to-serotonin ratio (DA:5HT)^28^. In these colonies, there was no correlation at the phenotypic level between colony sensitivity of foraging activity to humidity and average forager brain DA:5HT^28^, suggesting there may be other sources of molecular variation, apart from those that influence neurotransmitter titer, associated with behavioral variation. To characterize this molecular variation, we used RNA-seq to profile gene expression in the brains of individual foragers (Methods, *N* = 85 brains from *N* = 9 colonies), and then used supervised and unsupervised statistical approaches to discover transcriptomic patterns within and among colonies (Methods), their association with colony phenotypes, and evolutionary constraints on those patterns.

We test four primary hypotheses. First, we hypothesized that transcriptomic variation among the foragers would reflect a signature of colony identity, owing to shared genetic variation, maternal effects, and colony environment. Second, we hypothesized that the expression patterns of functionally relevant subsets of genes would be associated with variation in colony-level traits. Third, we hypothesized that coexpression networks would be enriched in neurophysiologically relevant functions and be associated with colony trait variation. Fourth, we hypothesized that genes with trait-associated expression, or high coexpression module centrality, would show unique patterns of evolutionary constraint, indicating an association between transcriptional regulation and selection pressure.

Taken together, our analyses show that the gene expression profiles of forager brains strongly vary among colonies of red harvester ants, and may reflect functional differences in neurophysiology among colonies that are related to neurotransmitter signaling and metabolism. We found that forager brain gene expression patterns were more similar among nestmates than non-nestmates. There was also substantial expression variability within a colony, perhaps owing to individual experience or temporal polyethism. We found that across the genome, expression patterns were correlated, both positively and negatively, with colony traits, and that genes more correlated with colony traits were enriched in various functional categories related to neural signaling. Neurotransmitter receptors as a category were significantly correlated in their expression pattern with colony sensitivity of foraging activity to humidity. Gene coexpression analysis identified 11 modules of loci with coordinated expression patterns across colonies. Coexpression modules identified from contemporary transcriptomic data were functionally enriched, had expression significantly correlated with colony traits, were differentially utilized among colonies, and had significantly divergent patterns of coding sequence constraint compared with the rest of the genome. These transcriptomic results show that gene expression and coexpression variation among colonies, especially related to biogenic amine signaling pathways, are associated with differences among colonies in behavior. Loci with expression patterns more central to coexpression modules are more correlated with trait variation among colonies, and also significantly diverge from the genomic background in protein-coding sequence constraint, suggesting a distinct evolutionary role for loci with colony-specific and behaviorally associated expression patterns.

## Results

### Colony variation in forager brain gene expression

**Fig. 1:**
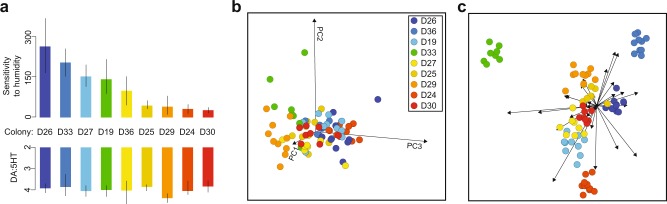
Colony variation in collective behavior, physiology, and forager brain gene expression. **a** Colony traits for the nine colonies profiled in this study. The top *Y* axis reflects colony sensitivity of foraging activity to humidity, in units of the number fewer foraging trips made per degree decrease in daily humidity (Methods). The bottom *Y* axis is forager brain dopamine-to-serotonin ratio (Methods), a measure of colony variation in neurophysiology. Bars are group means ± s.e.m. **b** The axes are the first three principal components of the single-forager brain transcriptome, capturing 37% of variation among samples. Each point is a single-forager brain transcriptomic sample, the point of the color represents colony. **c** Linear Discriminant Analysis on first 30 PCA dimensions (77% of variation). A point is a single-forager brain transcriptomic sample, the point of the color represents colony.

First, we found that colony-specific factors influence forager transcriptomes. There is also considerable transcriptomic variation among nestmates. To characterize colony differences from the transcriptomic data, we used dimensional reduction techniques. Principal component analysis (PCA) showed clear stratification by colony along the first three PC axes (Fig. 1b), as well as considerable statistical variation among individuals within colonies (Supplementary Fig. 1). Linear discriminant analysis showed transcriptomic differentiation among colonies along several principal component dimensions (Fig. 1c). A distance-based network analysis also demonstrated clustering by colony; samples most similar to one another tended to be nestmates (Methods, Supplementary Fig. 2). These results support the notion that aspects of individual’s transcriptomes are colony-specific, potentially driven by biological factors such as genetic diversity, microenvironment, or colony traits.

### Gene expression association with colony trait variation

**Fig. 2:**
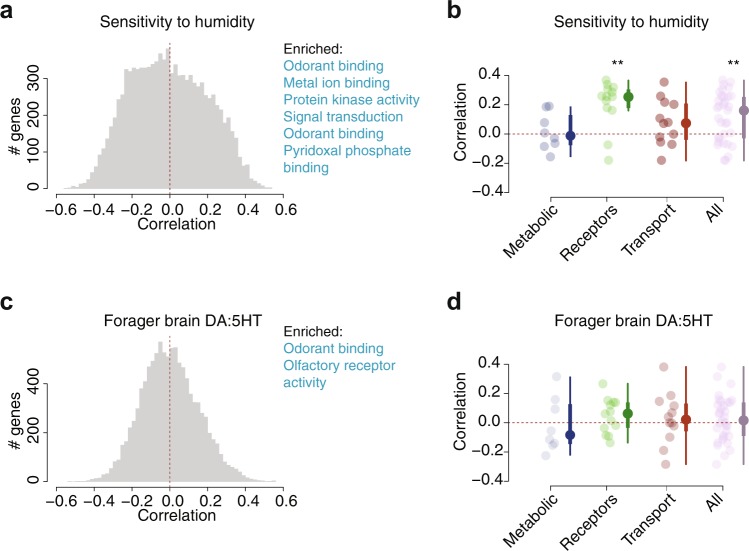
Gene expression correlations with colony behavior and physiological traits. The distribution of correlation coefficients are shown transcriptome-wide **a**, **c** and for candidate loci lists **b**, **d**, between colony traits and forager brain gene expression (*N* = 85). **a** Histogram of correlation coefficients between the expression of each gene in the *P. barbatus* transcriptome and sensitivity of foraging activity to humidity. **b** Expression correlation with colony sensitivity of foraging activity to humidity for the different candidate loci categories (**P < 0.001; one-sample *t* test). **c** Transcriptome-wide expression correlations with colony average forager brain dopamine-to-serotonin ratio. Bar color reflects the top (blue) 50% of correlation values used for the omnibus gene ontology enrichment test. The bottom 50% is not highlighted since no significant enrichments were detected. **d** Correlation with forager brain dopamine-to-serotonin ratio (DA:5HT) for the different candidate locus groups. (no tests with *P* < 0.01; one sample *t* test).

Next, we explored the extent to which the gene expression variation among colonies was associated with two colony traits: colony sensitivity of foraging activity to humidity and colony average forager brain DA:5HT (Fig. 2a, c, Supplementary Table 1). Phenotypes were quantified at the colony level (*N* = 9 colonies), whereas gene expression was profiled at the single-forager level (*N* = 8–10 foragers per colony, *N* = 85 libraries total). First, we quantified how gene expression variation within and among colonies was linearly associated with colony-level phenotypic variation. Expression-trait correlation values calculated with colony average expression levels were consistent with correlation values that take nestmate variation expression into account (Supplementary Fig. 3a, b, *p* < 0.00001, Pearson *r* = 0.926 with *R*^2^ = 0.858 for colony sensitivity of foraging activity to humidity; *p* < 0.00001, Pearson *r* = 0.878 with *R*^2^ = 0.77 for colony average DA:5HT). For both traits, the genome-wide correlation coefficients were roughly symmetric and centered around zero (Supplementary Fig. 3c, d). Many genes showed colony-specific expression patterns (e.g., with F statistics showing greater variation among colonies than within colonies). However, only a fraction of genes with colony-specific expression variation have linear associations with trait variation among colonies. To summarize the extent to which expression at each locus was associated with the two colony traits under study, we used the derive a summary coefficient for trait correlation. This value, which ranged from ~ −0.5 to 0.5 (Fig. 2a, b), captures how the expression of each gene is linearly associated with variation among colonies in the two traits.

### Functional analysis of trait-associated expression patterns

Gene ontology (GO) enrichment analyses indicated that loci that were increasingly correlated with the sensitivity of foraging activity to humidity were enriched for involvement in signal transduction (GO:0007165), protein kinase activity (GO:0004672), transmembrane receptor signaling (GO:0004888), and postsynaptic membrane localization (GO:0045211) (Supplementary File 1). Loci with expression increasingly correlated with the DA:5HT in forager brains were enriched in GO terms related to odorant binding (GO:0005549) and olfactory receptor activity (GO:0004984) (Supplementary File 2). There were no significantly enriched GO terms for genes with expression negatively correlated with colony average DA:5HT in forager brains.

We complemented the transcriptome-wide tests for expression-trait correlations with interrogations of gene lists known to be important for insect behavior: metabolic enzymes, biogenic amine SLC-family transporters, and G-protein coupled receptors of neurotransmitters (GPCRs)^16,38,39^ (Methods). We calculated the correlation of each candidate gene with the sensitivity of foraging activity to humidity and average forager brain DA:5HT, and then asked whether the average correlation of each list of loci was significantly different from zero for either of the two colony traits. The expression of biogenic amine receptors as a list was significantly positively correlated with sensitivity of foraging activity to humidity (Fig. 2b, two-sided one-sample *t* test for mean ≠ 0, *df* = 12, *t* = 4.62, *p* = 0.00059). No categories displayed an overall correlation with average forager brain DA:5HT (Fig. 2d, all tests are two-sided one-sample *t* test for mean ≠ 0: metabolic loci, *df* = 7, *t* = −0.212, *p* = 0.84; receptors, *df* = 12, *t* = 1.46, *p* = 0.17; transporters, *df* = 10, *t* = 0.59, *p* = 0.57). There were several interesting single-locus expression correlations from candidate gene lists that did not show an overall correlation, though no locus was a statistical outlier (scatterplots in Fig. 2b, d). First, though metabolic enzymes as a group did not differ from 0 in their average correlation with sensitivity of foraging activity to humidity, the expression of tyrosine hydroxylase (LOC105423652) was the candidate enzyme positively correlated with colony sensitivity to humidity (Pearson *r*^2^ = 0.18, *N* = 85). Tyrosine hydroxylase is the rate-limiting enzyme in dopamine biosynthesis in animals^40^ and previous experiments in *P. barbatus* showed that an increase in forager brain dopamine led to an increase in foraging activity^28^. Second, although on average the category of transporters had near-zero correlation with average forager brain DA:5HT, the transporter with the highest positive correlation to this ratio was the *P. barbatus* homolog of the *white* (LOC105432124) locus (Pearson *r*^2^ = 0.382, *N* = 85). In *Drosophila melanogaster*, *white* loss of function flies have reduced head dopamine levels^41^ and altered social behavior^42^. This could imply a conserved role for *white* across insects; perhaps, increased transporter expression results in increased (relative) brain dopamine titers via altered neuronal efflux of dopamine or related metabolites.

### Functional analysis of gene coexpression networks

**Fig. 3:**
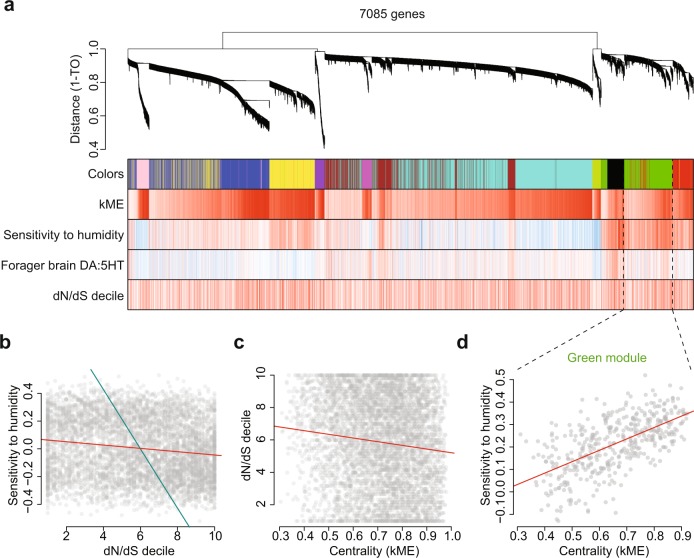
Summary of weighted gene correlation network analysis (WGCNA). **a** Dendrogram of hierarchically clustered genes, based on expression levels. “Leaves” along “branches” represent individual loci. The *Y* axis represents network distance as determined by 1 – topological overlap (TO), values closer to 0 indicate greater similarity of expression profiles between samples. The first color band denotes the coexpression module assignments for the loci directly above. Modules are named by color. The second color band represents Pearson correlations between gene expression profiles and module eigengenes (kME); the darker the shade of red, the closer the correlation value is to 1. The third and fourth color bands represent Pearson correlations between gene expression profiles and colony-level traits; sensitivity of foraging activity to humidity and forager brain dopamine to serotonin (DA:5HT) ratio, where darker the color of red, the closer the correlation value is to 1 and −1. The final color band represents the dN/dS decile for loci in the dendrogram such that darker the shade of red, the lower the dN/dS decile is for that gene (WGCNA Methods). **b** Correlation between gene expression level and sensitivity of foraging activity to humidity (*Y* axis) for individual loci (dots), plotted against the dN/dS decile (*X* axis) for the same loci. All 7085 loci in the dendrogram are shown. The red line is the best-fit linear regression and the blue line represents the regression slope among the 11 coexpression modules of module average dN/dS vs. average expression correlation with the sensitivity of foraging activity to humidity. **c** Relationship between module centrality of each locus (*X* axis) and dN/dS decile (*Y* axis). **d** Correlation between gene expression level and sensitivity of foraging activity to humidity (*Y* axis) for individual loci (dots) in the “green” coexpression module, plotted against their module centrality (*X* axis, expression correlation with green module eigengene). The red line is the best-fit linear regression. Genes that are more central to the module tend to have stronger correlations with colony behavioral traits (Pearson *r* = 0.52, *p* < 3e-42).

To test the third hypothesis, that gene coexpression networks would be associated with functional roles and colony trait variation, we used Weighted Gene Coexpression Network Analysis (WGCNA)^43^. There were 11 modules of loci with expression significantly correlated with both sensitivity of foraging activity to humidity and average forager brain DA:5HT (Supplementary Table 2). The coexpression modules were enriched for a variety of neurophysiological processes (Supplementary File 3: 261 enrichments summed over the 11 modules). We asked whether these gene expression modules were differentially utilized across colonies and associated with the two colony traits (Fig. 3a) (Methods)^8,44^. There was evidence that the modules captured a shared transcriptomic architecture: the extent to which a module’s expression was correlated with sensitivity of foraging activity to humidity was significantly associated with forager brain DA:5HT (*N* = 11, Pearson *R*^2^ = 0.436, *p* = 0.027).

### Gene coexpression network use and colony traits

Differences among colonies in the utilization of functionally coherent coexpression modules were associated with differences among colonies in behavior. We computed the first principal component of each coexpression module to summarize within-module variability (a.k.a., module “eigengenes” (MEs)), and compared the expression of modules among colonies (Supplementary Fig. 4). Overall, loci more central to coexpression modules were more correlated with colony traits (e.g., Fig. 3b, Supplementary Fig. 5). In all 7 of the 11 modules with a significant regression, loci with higher module centrality have a significantly higher correlation with sensitivity of foraging activity to humidity (Supplementary Fig. 6). Some of the largest differences in coexpression module use mapped well to the two colony traits. For example, the expression of the “black” and “green” MEs were significantly increased in colony D27, a colony with high sensitivity of foraging activity to humidity, relative to colonies D24 and D30, which have lower sensitivity of foraging activity to humidity (Supplementary Fig. 5). Interestingly, loci in these modules had the strongest average expression correlations to colony sensitivity of foraging activity to humidity (Supplementary Table 2), and were enriched in GO terms including GPCR activity and cyclic nucleotide-related signaling pathways (Supplementary File 3). This suggests that colony differences In behavior are strongly associated with differential utilization of functionally enriched coexpression networks. Further work is needed to investigate this.

### Evolutionary patterns and trait-associated expression

At last, we used phylogenetic comparative methods to test the hypothesis that genes with trait-associated expression or high coexpression module centrality would show unique patterns of evolutionary constraint. We tested how forager brain gene expression patterns were associated with the genomic signature of protein-coding sequence constraint over evolutionary time (~ 10–120 million years), as measured by the dN/dS statistic^44–47^ (Methods). We first tested the relationship between dN/dS and the extent to which a gene had trait-associated expression patterns. The results show that genes more correlated with colony sensitivity of foraging activity to humidity, and with forager brain dopamine to serotonin, are evolving under increased coding sequence constraint (GLM, *p* < 0.00001 for both traits (Supplementary File 4). This result held even after accounting for the effect of known factors that influence dN/dS, such as expression level and variability (Supplementary File 4).

It appears that loci more central to coexpression modules, and more correlated with colony traits, are evolving under stronger protein sequence constraints than the rest of the genome. Genes in coexpression modules had significantly lower dN/dS than those not in coexpression modules (*p* = 1e-14, Supplementary Fig. 6), suggesting that genes with more coordinated transcriptional patterns are evolving under increased protein sequence constraints. Further, gene coexpression modules differed significantly in average dN/dS values, both from one another and from the genomic background: 5 of the 11 coexpression modules consisted of loci evolving under significantly decreased coding sequence constraint, whereas one module consisted of loci that are evolving under significantly increased coding sequence constraint (two-sided one-sample *t* test, *p* < 0.005, Supplementary Table 2, Supplementary Fig. 7). Across coexpression modules, coding sequence constraint was correlated with the extent to which the module was associated with sensitivity of foraging activity to humidity (*R*^2^ = 0.799, *p* < 0.0001, *N* = 11, blue line in Fig. 3b), but not the ratio of dopamine to serotonin in forager brains (*p* > 0.2, *N* = 11). Within modules, genes that are more central to modules tend to have stronger correlations with colony behavioral traits (Fig. 3d, Supplementary Fig. 8, ANOVA, *n* = 7,085 loci, *p* < 3e-42; linear regression *p* = 1.5e-18, GLM *p* < 1e-5, Supplementary File 4). The positive association between coexpression centrality and degree of coding sequence constraint persists even after accounting for locus-level descriptors such as expression level (average transcripts per million for the locus) and degree of variability among colonies (the variance in expression among all samples) (ANOVA, *p* < 2.2e-16, Supplementary Fig. 9, Supplementary Fig. 10, Supplementary File 4). For almost all modules, when compared separately with most species, there is a negative relationship between coexpression centrality and dN/dS (Supplementary Fig. 10). This indicates a positive association between centrality in a coexpression module and the degree of protein-coding sequence conservation over evolutionary time. A similar relationship between gene-level coexpression module centrality and coding sequence constraint has been observed in honey bees^48^, other ant species^45^, and some plant species^49^. Loci under tighter transcriptional regulation may be under increased sequence constraint owing to multi-tissue pleiotropy^48^ or increased deleterious effects of dysfunction in regulatory genes rather than secretory proteins^50^. Consistent with this, we have found evidence of increased sequence constraint on highly expressed genes, and genes more central to coexpression networks.

## Discussion

Here we used RNA sequencing on red harvester ant forager brains and found that colonies have distinct gene expression signatures, and that nestmates resemble one another in brain expression patterns. Genes with expression increasingly associated with colony collective behavioral and physiological traits were enriched in functional terms related to neural signaling. Genes with expression patterns more central to coexpression modules tended to be more correlated with colony traits and also evolving under increased coding sequence constraint.

Here, we considered the association between single-forager brain gene expression and variation among colonies. We did not measure behavioral variation among nestmates within a colony, which might be associated with age or experience. It would be interesting to learn how such variation within a colony in the brain gene expression of workers, as well as differences among workers in behavior, is associated with colony traits. We considered the transcriptome of the whole brain, which is a complex tissue composed of many heterogeneous cell types, and our results confirm that natural behavioral variation among colonies is linked to broad-scale differences in brain physiology. More-pronounced physiological variation among colonies would probably be detected if biogenic amine levels were quantified at the brain-region scale, or if gene expression patterns were quantified at the single-cell scale^51^. It would be interesting to examine the association between variation in the traits measured here and colony fitness, and relatedness among colonies, as in previous work^9,52^. However, we do not have fitness estimates for this set of colonies. This type of analysis could elucidate the relationship between colony success and molecular variation, for example, by finding expression-modulating genetic variants that show signatures of selective sweeps, or evidence of shared colony microenvironment.

By expanding the scope of evolutionary transcriptomics beyond reproductive and behavioral differences among nestmates^7,53^, we found that contemporary transcriptomic variation among ant colonies in a natural setting are enriched in loci related to biogenic amine signaling and metabolism. Variation among colonies in worker neurophysiology may underlie ecologically important variation among colonies in collective behavior, and thus influence colony reproductive success, through alterations to worker sensitivity to interactions or environmental conditions^15,18^. Loci with expression patterns correlated with colony variation in behavior appear to be evolving under increased protein-coding sequence constraint, suggesting that ant colony behavior can evolve through non-coding and epistatic changes to the genome, as well as through coding sequence changes at longer time scales. Gene expression differences among colonies of *P. barbatus* could be influenced by heritable factors, and thus could reflect the raw material shaped by past and current selection acting on colony behavior.

## Methods

### RNA sequencing

Foragers were collected from nine *P. barbatus* colonies near the site of a long-term study of this species in Rodeo, New Mexico^52^. The same colonies were used in previous behavioral observations and pharmacological experiments^28^. In previous work, we measured for each colony (1) the sensitivity of foraging activity to humidity as the reduction in foraging trips made per day per % decrease in daily relative humidity, and (2) average forager brain DA:5HT ratio, measured with high-performance liquid chromatography^28,54^. Foragers were identified when leaving the nest mound walking in a straight line, carrying nothing, in a direction used by other foragers that day^28^. Ants were collected by aspiration and placed directly into liquid nitrogen. Foragers were collected from all colonies within 2 hours on the same morning (9/4/2017). The same collection of ants was used for gene expression quantification in this analysis as for the measurements of forager brain DA:5HT from these colonies^28^.

For RNA sequencing, single-forager brains were dissected out in a fresh buffer of cold RNAlater and placed into Trizol on dry ice^55^. Total RNA was extracted from whole forager brains, and RNA-seq libraries were prepared by Novogene using the following protocol. From each single brain, <1 µg of RNA was used for cDNA library construction using the NEBNext Ultra RNA Library Prep Kit for Illumina (cat# E7420S, New England Biolabs, Ipswich, MA, USA) according to the manufacturer’s protocol. rRNA was removed using the Ribo-Zero kit. The mRNA was fragmented randomly by adding fragmentation buffer, then cDNA was synthesized by using mRNA template and random hexamers primer, after which a custom second-strand synthesis buffer (Illumina), dNTPs, RNase H and DNA polymerase I were added to initiate the second-strand synthesis. Next, after a series of terminal repair, ligation, and sequencing adaptor ligation reactions, the double-stranded cDNA library was completed through size selection and PCR enrichment. The resulting 250–350 bp insert libraries were quantified using a Qubit 2.0 fluorometer (Thermo Fisher Scientific, Waltham, MA, USA) and quantitative PCR. Size distribution was analyzed using an Agilent 2100 Bioanalyzer (Agilent Technologies, Santa Clara, CA, USA). Qualified libraries were sequenced on an Illumina HiSeq 4000 Platform (Illumina, San Diego, CA, USA) using a paired-end 150 run (2 × 150 bases). Around 20 M raw reads were generated from each library. Sequencing outcomes per library are available in file “NovogenePbarSeqQC.xlsx”. Ten brains from each of the nine colonies were sent for sequencing. Five samples were discarded before the library preparation phase, resulting in *n* = 85 high-quality single-forager brain transcriptomes.

### Bioinformatics

The *P. barbatus* genome and transcriptomic resources were downloaded from NCBI (GenBank accession: GCF_000187915.1^56^). Previous brain-specific RNA-seq data were used to improve gene models in this NCBI assembly^28^ (Bioproject: PRJNA277638).

Candidate gene lists were constructed for three categories of *P. barbatus* neurotransmitter-related loci: metabolic enzymes, GPCR receptors, and transporters. The list of candidate biogenic amine receptors was found by querying dopamine, histamine, octopamine, tyramine, and serotonin receptors from *Drosophila* against the *P. barbatus* proteome. The list of transporters was generated by finding all *P. barbatus* homologs of neurotransmitter receptors identified in *Drosophila*^57^. In all cases, the *Drosophila melanogaster* protein sequence was used as a BLASTP query against the *P. barbatus* genome and an *E* value cutoff of 0.01 was used. The list of biogenic amine metabolic enzymes was generated by finding all *P. barbatus* homologs of the canonical invertebrate biogenic amine-metabolizing enzymes^16,38,39^. To avoid user bias in the construction of candidate gene lists, the lists were finalized before calculating associations with colony traits, and all relevant genes from recent review papers were used as queries against the *P. barbatus* proteome.

RNA-seq read quality was assessed using MultiQC^58^. Adapter sequences were removed using cutadapt^59^ with standard settings. Reads were aligned to the *P. barbatus* transcriptome using STAR^60^ allowing for two sequence mismatches per read (to accommodate potential genetic divergence between the sampled populations and the reference). Gene-level counts were extracted using Rsubread^61^ and the *P. barbatus* annotation GCF_000187915.1_Pbar_UMD_V03_genomic.gff^56^. Raw counts were converted to transcripts per million (TPM) for downstream analyses. We controlled for potential sequencing batch effects in R with ComBat^62^, using sequencing run, RNA concentration (via qubit measurement), and library number as potential effects. Gene expression characteristics such as overall expression level and extent of variability among samples are known to be associated with distinct evolutionary patterns. For use in downstream analyses, for each locus we calculated the median expression level (Median TPM), and expression variability (Standard deviation among all samples).

PCA and linear discriminant analysis (LDA) were carried out in Orange 3.20 (workflow available). A PCA was calculated on an expression matrix where columns correspond to samples, rows correspond to loci in the *P. barbatus* transcriptome, and cell values were expression values in TPM. Thirty principal component dimensions were retained for the LDA, with colony used as a stratifying factor.

We tested for correlations of gene expression level with two measures of colony traits, described above: (1) sensitivity of foraging activity to humidity, and (2) forager brain DA:5HT. Correlations were calculated in two different ways: a measure of a colony trait with colony average expression regression (*N* = 9 points), and a measure of a colony trait with per-sample expression level (*N* = 85 points). The coefficients of expression-trait correlations were in good agreement in the two methods (Supplementary Fig. 3, *p* < 0.0001 for both traits). We performed downstream quantitative analysis with the *N* = 85 single-brain level regression slope estimates, because correlation coefficients calculated with the single-forager expression levels had more accurate estimates of slope and variance owing to the larger sample size, and better captured the subset of genes that had stable linear expression patterns associated with colony trait variation (Supplementary Fig. 3).

GO term analysis used an omnibus *p* value approach to find functional enrichment of loci that were correlated with traits. First, for each gene we obtained associated GO terms using GO FEAT^63^. To derive an omnibus *p* value for GO term enrichment, we calculated gene set enrichment statistics for the top 50–99% of the loci with expression correlated to colony traits using a Fisher’s exact test, then repeated the test by restricting the set of associated loci with increased stringency by 5% steps (e.g., top 50% of loci, then top 45%, top 40%, etc.) and combined the resulting *p* values using Fisher’s method. We used a cutoff of post-correction omnibus *p* value of < 0.05 for GO term enrichment and did not consider GO term enrichments when a given gene set had fewer than 10 annotated sequences. The resulting omnibus *p* values should reflect gene sets that are consistently enriched in the tails of the colony trait correlation distributions at varying levels of stringency, as opposed to single cutoffs as is commonly used. This process should thus filter out potential false-positive associations and highlight GO terms that strongly vary with colony traits.

### WGCNA analysis

#### Data pre-processing and batch correction

All data pre-processing and coexpression network analysis was done in R^64^. Raw TPM values were processed to remove genes with no variance across samples, low median expression (TPM < 0.5), or no expression in more than one-third of the samples. Next, gene expression values were log-transformed, then outlier genes and samples were removed in an iterative process, as described previously^43,65,66^. In brief, gene expression values more than three standard deviations (SD) from the mean for that gene across all samples were masked out, and samples with a mean inter-sample correlation more than two SD from the overall mean were removed. This process was repeated until no more expression values or samples exceeded these thresholds, and resulted in the removal of 33 samples and 775 genes, leaving 11,641 genes across 52 samples representing six colonies (D19: *n* = 8; D24: *n* = 10; D25: *n* = 10; D27: *n* = 9; D29: *n* = 8; D30: *n* = 7) for further analysis. Samples were quantile normalized, then the effects of the sequencing run on average gene expression were corrected using ComBat^62^, with colony as a biological covariate.

#### Network construction

WGCNA was performed as described, in the R WGCNA library^43,65–67^. Signed Pearson correlations were computed for all gene–gene pairs to generate a symmetric correlation matrix, which was transformed using a power function ((1+correlation)/2)^*β*^) to form the adjacency matrix of network connection strengths. *β* was determined empirically using the scale-free topology criterion (signed network: *β* = 12^68^). Next, a topological overlap (TO) matrix was computed based on the adjacency matrix and average linkage hierarchical clustering was performed using 1–TO as the distance metric^69^. Modules were defined using a dynamic tree cutting algorithm to prune the resulting dendrogram^70^, and labeled by arbitrary colors underneath the dendrogram. To study module composition, MEs were defined as the first principal component of each module, effectively summarizing the expression variability within modules. MEs were used to quantitatively relate gene coexpression patterns to phenotypic traits and construct ME correlation networks to study higher-order relationships among the modules. Eigengene-based connectivity (kME) was defined as a gene’s correlation with the ME, quantifying the extent to which its expression profile conformed to the largest source of variability within the module.

#### Enrichment for densely interconnected modules

An iterative filtering process was performed to enrich the final network with modules composed only of the most densely interconnected genes^43,66,70^. First, the soft threshold for constructing a signed weighted coexpression network was determined with the scale-free topology criterion applied to all genes. Then, a preliminary network was constructed using default module definition (dynamic tree cutting) settings, except for a minimum module size of *n*  = 80 genes, to ensure suitable power for downstream module enrichment tests. The average TO within each module was defined as the module density, which was then compared with the density of 10,000 pseudo-modules of the same size that were generated by randomly selecting genes from the network. A *p* value for the density of each module was defined as the number of pseudo-module densities greater than the actual density, divided by 10,000. Genes in modules with *p* > 0.01 and gray background genes were removed. The network was rebuilt with the remaining genes, and the process was iterated until all modules passed the density test and there were no more gray genes, leaving 7085 genes in the final network. To confirm the efficacy of the additional filtering for ensuring module robustness, we computed module quality statistics using the WGCNA function modulePreservation^70^. Typical module preservation statistics were used to evaluate the preservation of modules in test networks created by randomly permuting the actual gene module assignments. These statistics were interpreted as indicators of module density and separability (distinctness of modules from others in the network), i.e., module quality. Averaged across many permutations of the original data, module quality statistics were indicative of module robustness and reproducibility. Summary scores of *Z* > 10 were interpreted as strong evidence of densely interconnected, distinct, reproducible modules, and modul *Z* scores ranged from 11.23 (green module) to 63.48 (pink module). Modules ranged in size from 108 to 2182 genes (median = 355 genes).

### dN/dS estimation and analysis

dN/dS is a summary statistic that quantifies the degree to which each protein-coding genomic locus is constrained by purifying selection acting on its translated sequence. A low dN/dS value represents a strong history of purifying selection, or high sequence constraint, whereas a high dN/dS value can represent either relaxed selection or positive selection for a novel trait. The dN/dS values were calculated using the “orthologr” package^71,72^. Orthologs were computed between the predicted proteome of *P. barbatus* and six other species: the honey bee *Apis mellifera*^73^, plus five ant species: *Harpegnathos saltator*^74^, *Ooceraea biroi*^75^, *Monomorium pharaonis*^45^, *Linepithema humile*^76^, and *Camponotus floridanus*^74^. Homologs were identified with a reciprocal best BLAST hit approach at a cutoff *E* value of 1E-5. dN/dS was calculated with the method of Comeron^77^. For each species, every non-missing dN/dS value was then recoded as its decile value (e.g., lowest 10% of dN/dS values recode to 1, highest 10% of dN/dS values recode to 10). The decile transformation facilitates comparisons across species of various evolutionary distances^71,72^.

For analyses involving the relationships between gene expression, dN/dS, and WGCNA module centrality, a subset of 5186 loci with an estimated dN/dS was constructed from the list of 7085 loci included in any of the 11 modules. For analyses involving raw TPM expression level as a predictor, we performed all regressions on all 5186 loci, as well as the subset of 5138 genes that were in the lower 99% of expression percentiles and lower 99% of expression variability.

We used a generalized linear model to consider how coexpression centrality and correlation with colony traits were associated with evolutionary coding sequencing constraint (dN/dS) after accounting for the covariates of expression level (Median TPM), and expression variability (standard deviation among all *n* = 85 samples). Full results for the models are provided in Supplementary File 4).

### Statistics and reproducibility

Statistical analysis was performed with the specific methods described in sections above. Nine colonies were chosen for RNA sequencing because these were the colonies with behavioral and physiological trait data. From each colony, we created RNA-seq libraries from 10 single-forager brains. The sequencing company (Novogene) completed library construction and sequencing for 85 of the 90 libraries. All biological samples were consumed in the RNA-seq library preparation process, and thus are no longer available. To ensure bioinformatic accuracy and reproducibility, all RNA-seq data were used for downstream analysis, except when samples or loci were pruned for specific tests and following standard protocols. RNA-seq data from each nestmate brains was considered as a biological replicate with respect to tests for colony variation in gene expression, and as an individual sample (with colony as sample metadata) for tests involving gene coexpression.

### Reporting summary

Further information on research design is available in the Nature Research Reporting Summary linked to this article.

## Supplementary information

Descriptions of Additional Supplementary Files

Supplementary Data 1

Supplementary Data 2

Supplementary Data 3

Supplementary Data 4

Reporting Summary

Supplementary Information

## Data Availability

All data are available on the Stanford Digital Repository at the Stanford Libraries, at the following persistent url: https://purl.stanford.edu/td277vn9006. Data for all Figures are included in the SDR archival site. Raw RNA-seq reads are available at BioSample: SUB5744886.

## Code availability

All relevant code and files are available on the Stanford Digital Repository at the Stanford Libraries at the following persistent url: https://purl.stanford.edu/td277vn9006.
