# Supplementary material for: Gene expression variation in the brains of harvester ant foragers is associated with collective behavior
Source: Commun Biol. 2020 Mar 5;3:100. doi: 10.1038/s42003-020-0813-8 (PMC7057964; doi:10.1038/s42003-020-0813-8)
Supplement: Supplementary file 1 — Descriptions of Additional Supplementary Files [file 42003_2020_813_MOESM1_ESM.pdf]

Descriptions of Additional Supplementary Files

Supplementary Data 1
Gene Ontology omnibus enrichment results for genes with expression patterns correlated with colony sensitivity of foraging activity to humidity.

Supplementary Data 2
Gene Ontology omnibus enrichment results for genes with expression patterns correlated with colony average forager brain dopamine to serotonin ratio.

Supplementary Data 3
Gene Ontology enrichment results for each coexpression module.

Supplementary Data 4
Statistical outputs for multivariate GLM testing the association between expression patterns (centrality, and correlation to colony trait variation) and locus-level descriptors (average expression level, expression variation among samples, and locus dN/dS).
