# Supplementary material for: Gene expression variation in the brains of harvester ant foragers is associated with collective behavior
Source: Commun Biol. 2020 Mar 5;3:100. doi: 10.1038/s42003-020-0813-8 (PMC7057964; doi:10.1038/s42003-020-0813-8)
Supplement: Supplementary file 6 — Reporting Summary [file 42003_2020_813_MOESM6_ESM.pdf]

nature research

Corresponding author(s): Deborah M Gordon

Last updated by author(s): Jan 17, 2020

Reporting Summary

Nature Research wishes to improve the reproducibility of the work that we publish. This form provides structure for consistency and transparency in reporting. For further information on Nature Research policies, see Authors & Referees and the Editorial Policy Checklist.

Statistics

For all statistical analyses, confirm that the following items are present in the figure legend, table legend, main text, or Methods section.

n/a	Confirmed	
☐	☒	The exact sample size (*n*) for each experimental group/condition, given as a discrete number and unit of measurement
☐	☒	A statement on whether measurements were taken from distinct samples or whether the same sample was measured repeatedly
☐	☒	The statistical test(s) used AND whether they are one- or two-sided<br>*Only common tests should be described solely by name; describe more complex techniques in the Methods section.*
☐	☒	A description of all covariates tested
☐	☒	A description of any assumptions or corrections, such as tests of normality and adjustment for multiple comparisons
☐	☒	A full description of the statistical parameters including central tendency (e.g. means) or other basic estimates (e.g. regression coefficient) AND variation (e.g. standard deviation) or associated estimates of uncertainty (e.g. confidence intervals)
☐	☒	For null hypothesis testing, the test statistic (e.g. *F*, *t*, *r*) with confidence intervals, effect sizes, degrees of freedom and *P* value noted<br>*Give P values as exact values whenever suitable.*
☒	☐	For Bayesian analysis, information on the choice of priors and Markov chain Monte Carlo settings
☐	☒	For hierarchical and complex designs, identification of the appropriate level for tests and full reporting of outcomes
☐	☒	Estimates of effect sizes (e.g. Cohen's *d*, Pearson's *r*), indicating how they were calculated

Our web collection on statistics for biologists contains articles on many of the points above.

Software and code

Policy information about availability of computer code

Data collection	Data collection related to colony traits is described in Friedman et al. 2018, iScience.<br>RNA-seq raw data and output files first presented in this article were collected as per the Methods section.
Data analysis	Data analysis Methods are described in the manuscript, and files are available at https://purl.stanford.edu/td277vn9006 .<br>ComBat and STAR were used for RNA-seq, WGCNA was used for coexpression analysis, R and the package orthologr were used for phylogenetic analysis and multivariate modeling.

For manuscripts utilizing custom algorithms or software that are central to the research but not yet described in published literature, software must be made available to editors/reviewers. We strongly encourage code deposition in a community repository (e.g. GitHub). See the Nature Research guidelines for submitting code & software for further information.

Data

Policy information about availability of data

All manuscripts must include a data availability statement. This statement should provide the following information, where applicable:

- Accession codes, unique identifiers, or web links for publicly available datasets
- A list of figures that have associated raw data
- A description of any restrictions on data availability

All data are available on the Stanford Digital Repository at the Stanford Libraries, at the following persistent url: https://purl.stanford.edu/td277vn9006 . Data for all Figures are included in the SDR archival site. Raw RNA-seq reads are available at BioSample: SUB5744886.

Field-specific reporting

Please select the one below that is the best fit for your research. If you are not sure, read the appropriate sections before making your selection.

☐ Life sciences ☐ Behavioural & social sciences ☒ Ecological, evolutionary & environmental sciences

For a reference copy of the document with all sections, see nature.com/documents/nr-reporting-summary-flat.pdf

Ecological, evolutionary & environmental sciences study design

All studies must disclose on these points even when the disclosure is negative.

Study description	This study characterizes patterns of forager brain gene expression variation within and among colonies of red harvester ants. From 9 colonies where colony traits had been quantitatively measured, we sequenced the brain transcriptome from N=85 foragers. WGCNA is used to construct gene coexpression networks, and dN/dS values are calculated to test for associations between evolutionary and expression-level statistics.
Research sample	85 foragers of red harvester ants (Pogonomyrmex barbatus), sampled from 9 colonies near Rodeo, New Mexico.
Sampling strategy	All foragers were collected within a 1 hour interval on the same morning. All colonies are from the same population and spatially located within a 100 meter by 100 meter area, near Rodeo, New Mexico, USA. The 9 colonies were chosen because they were the entire panel of species used in Friedman et al. 2018, no statistical power calculations were performed to choose the number of colonies.
Data collection	Samples were collected by DAF. Brains were dissected by DAF. RNA-seq library preparation and sequence was performed by Novogene Corporation (Sacramento, CA). Bioinformatics was performed by DAF, RAY, and ATH. Manuscript writing was performed by all 4 authors.
Timing and spatial scale	All foragers were collected within a 1 hour interval on the same morning in (September 4th 2017). All colonies are from the same population and spatially located within a 100 meter by 100 meter area, near Rodeo, New Mexico, USA.
Data exclusions	All 85 brain samples that produced successful RNA-seq libraries were used for transcript abundance quantification and expression correlation analyses. 33 samples were excluded from WGCNA analysis, the Methods used to exclude samples for coexpression analysis are descripbed in the text.
Reproducibility	No manipulations were performed in this manuscript. To ensure bioinformatic reproducibility, relevant data analysis/output files are available, and during the analysis the effect of algorithm parameters was explored (e.g. testing if number of mismatches allowed during read alignment, and testing whether GLM results were influence by the 1% of loci with highest expression level or expression variability).
Randomization	Randomization was not relevant to this study as no experimental manipulations were performed.
Blinding	RNA-seq library preparation and bioinformatics were performed blind to colony identity or traits.<br>All analysis of expression patterns and their association with colony traits was performed blinded to the colony traits.

Did the study involve field work? ☐ Yes ☒ No

Reporting for specific materials, systems and methods

We require information from authors about some types of materials, experimental systems and methods used in many studies. Here, indicate whether each material, system or method listed is relevant to your study. If you are not sure if a list item applies to your research, read the appropriate section before selecting a response.

Materials & experimental systems

n/a	Involved in the study	
☒	☐	Antibodies
☒	☐	Eukaryotic cell lines
☒	☐	Palaeontology
☒	☐	Animals and other organisms
☒	☐	Human research participants
☒	☐	Clinical data

Methods

n/a	Involved in the study	
☒	☐	ChIP-seq
☒	☐	Flow cytometry
☒	☐	MRI-based neuroimaging
