# Supplementary material for: Gene expression variation in the brains of harvester ant foragers is associated with collective behavior
Source: Commun Biol. 2020 Mar 5;3:100. doi: 10.1038/s42003-020-0813-8 (PMC7057964; doi:10.1038/s42003-020-0813-8)
Supplement: Supplementary file 7 — Supplementary Information [file 42003_2020_813_MOESM7_ESM.pdf]

Supplementary Information

Supplementary Figures

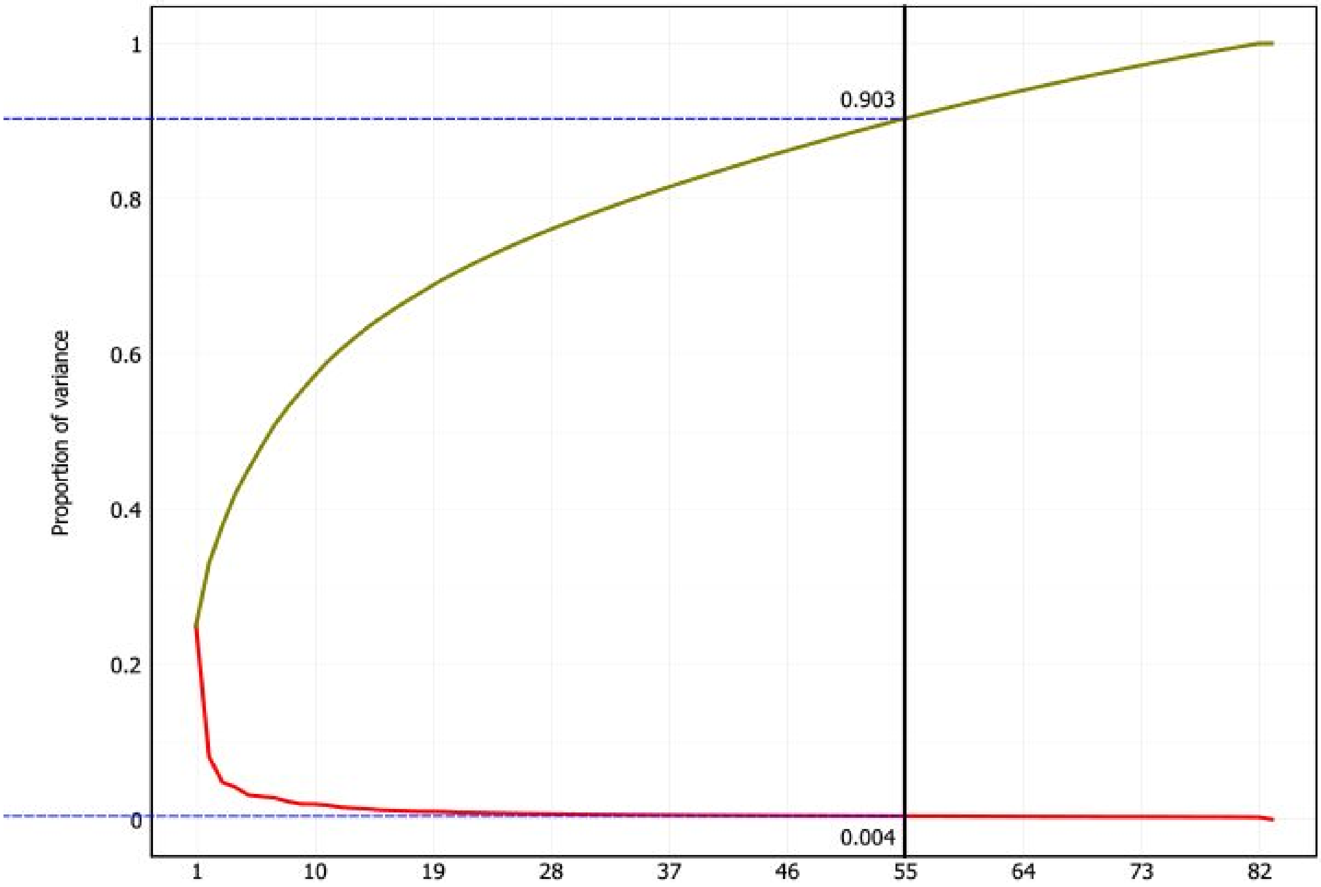

Supplementary Figure 1.

Principal Component Analysis curve reflecting the "Proportion of variance" explained by an increasing number of PC dimensions. The X-axis is the number of PC dimensions included in the analysis (whole transcriptome, N=85 single forager brains). The Y-axis is the proportion of statistical variation explained by that many PC dimensions, between 0 and 1. The black vertical line reflects that 55 PC dimensions explain 90% of the overall statistical variation.

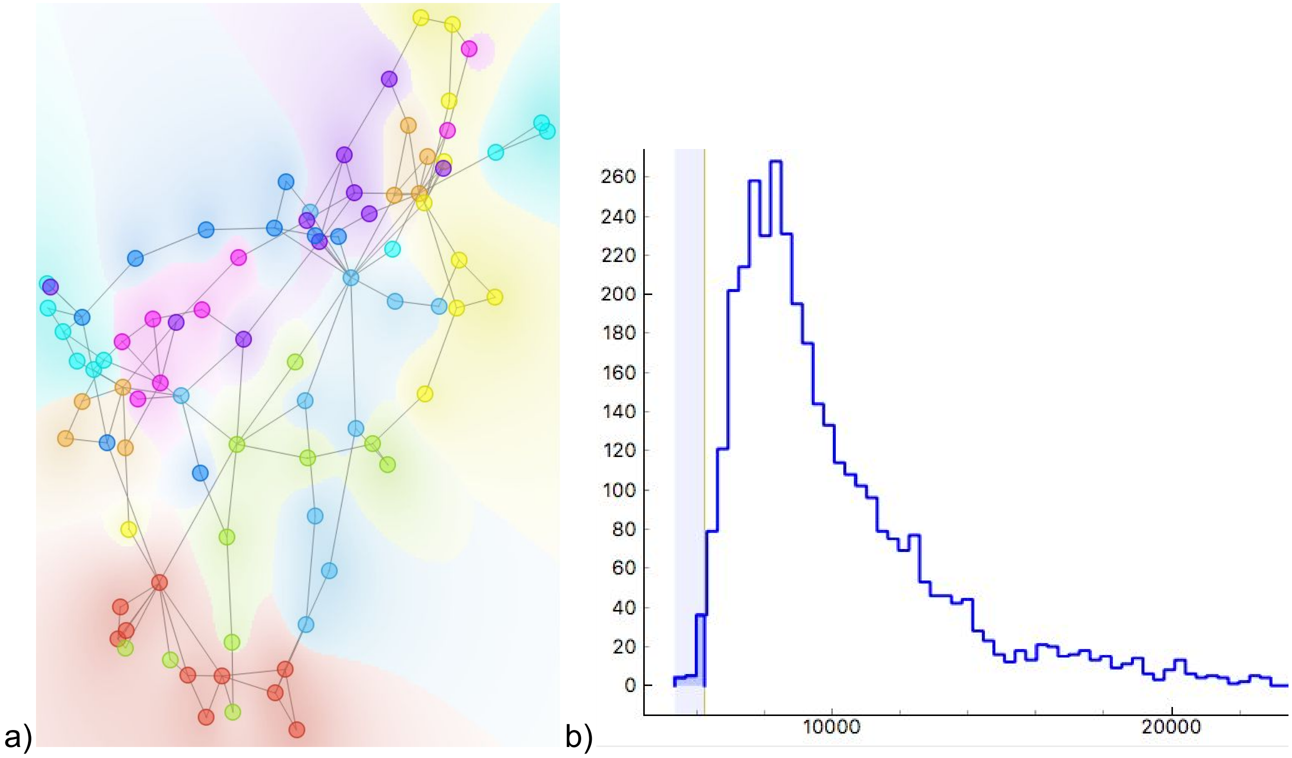

Supplementary Figure 2.

Topological analysis of transcriptomic similarity among samples a) Topological similarity network of single forager brain transcriptomes (nodes) colored by colony. Edges on the network were constructed as follows: All sample-sample distances were computed using a Manhattan distance metric on TPM expression of all loci. Edges were retained for sample-sample distances within the top 1% of all edge distances, as seen in shaded area of b) the histogram of all pairwise transcriptome-transcriptome divergences, in units of Manhattan distance, for N=85 single forager brain samples.

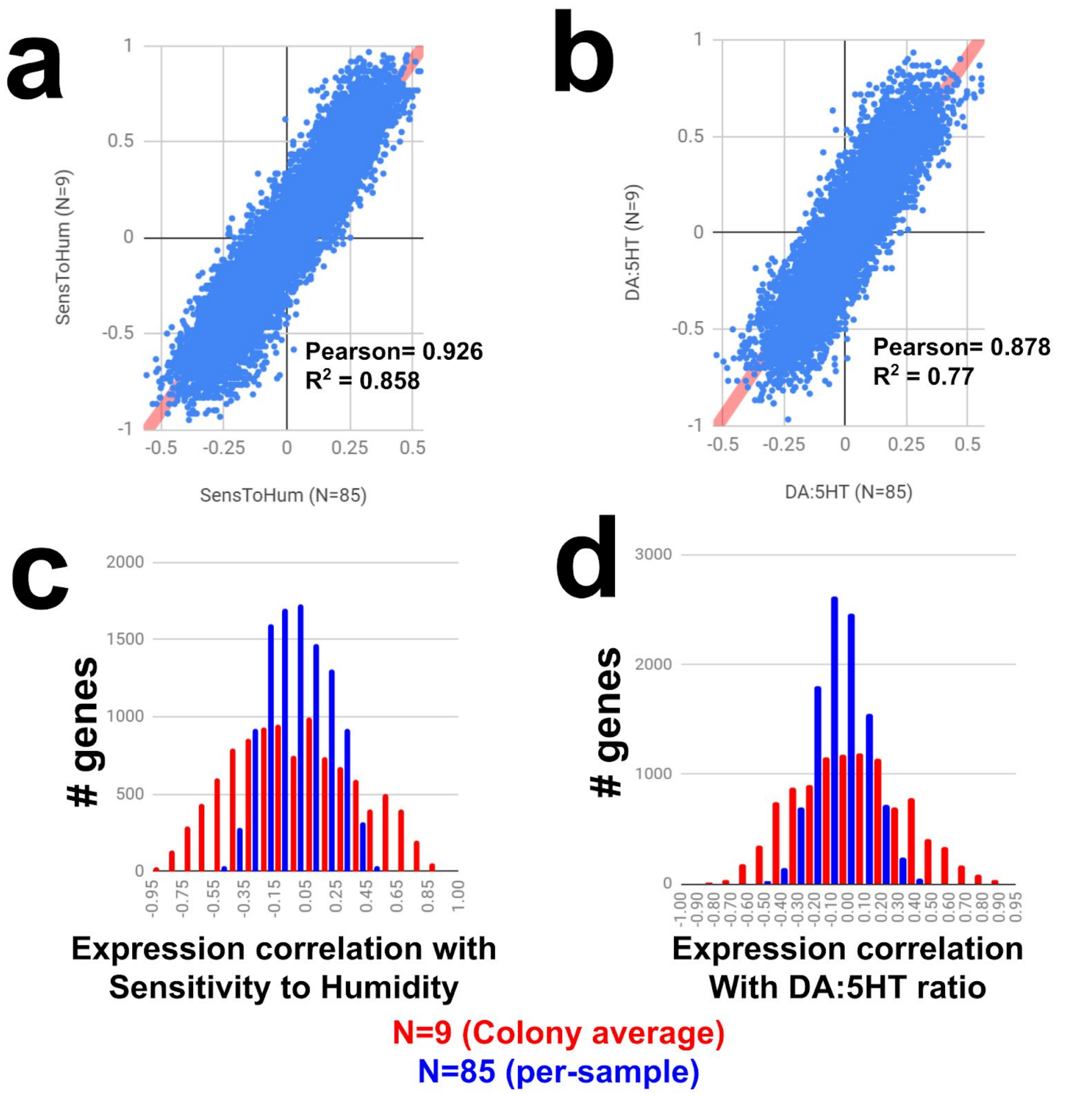

Supplementary Figure 3.

A and B) Relationship between expression~trait coefficients calculated with single forager brain expression levels (X-axes, N=85 samples from 9 colonies) or colony-level average expression levels (Y-axes, N=9 colonies with 7-10 individuals per colony). C and D) Histograms of expression correlation distributions for the two traits, calculated at the individual (blue) and colony (red) level.

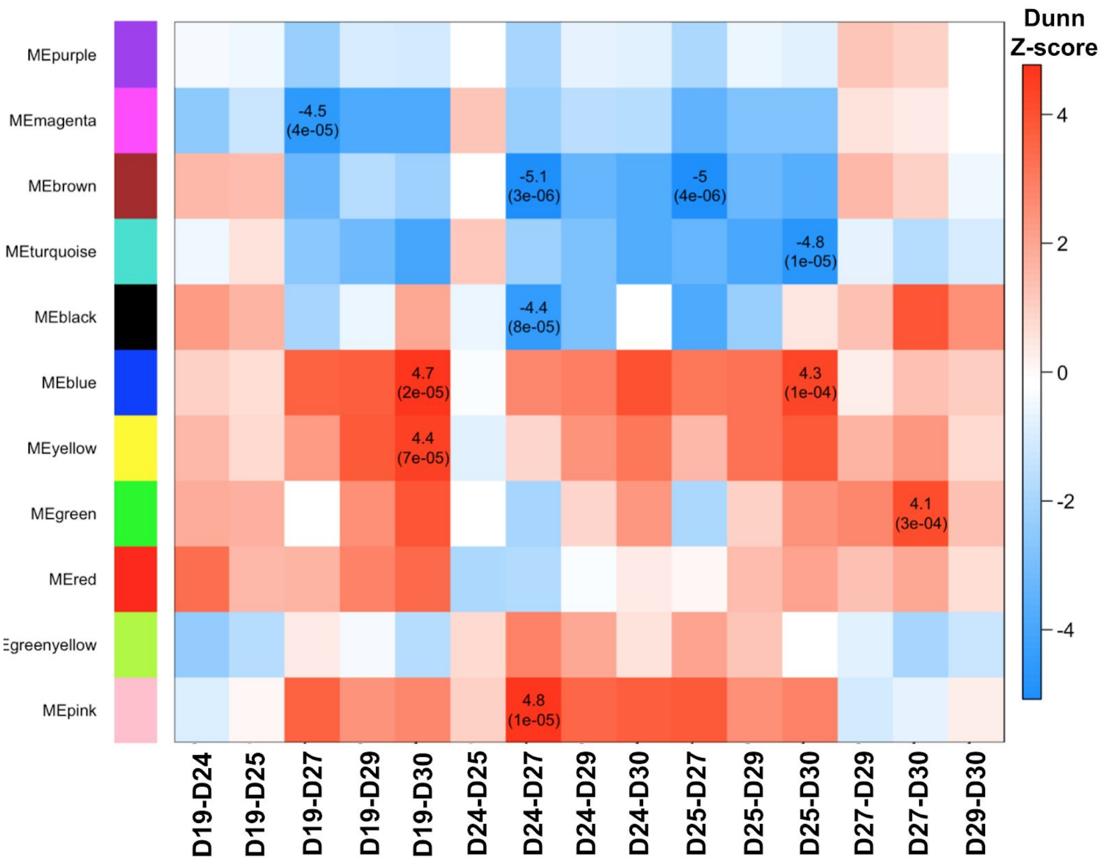

Supplementary Figure 4.

Heat map showing differential usage of coexpression modules across colonies.

Rows are module eigengene (MEs) expression patterns, defined as the first principal component of each module across pairwise colony contrasts (columns) using Dunn's test of multiple comparisons [1]. The results reflect variation among colonies in the average expression values of genes in a specific module, weighted by the variability in the module. Each pairwise comparison in Dunn's test is equivalent to a Wilcoxon-Mann-Whitney rank-sum test, and it can be considered a test for median difference that has the added feature of generating Z-statistic approximations of the actual rank statistics. Darker shades of red and blue correspond to more positive and negative Z-scores, respectively, and white corresponds to Z=0. For each ME, p-values were Bonferroni-corrected for 15 pairwise colony-colony comparisons, and only those that passed an additional Bonferroni-correction for 11 MEs are shown in the plot.

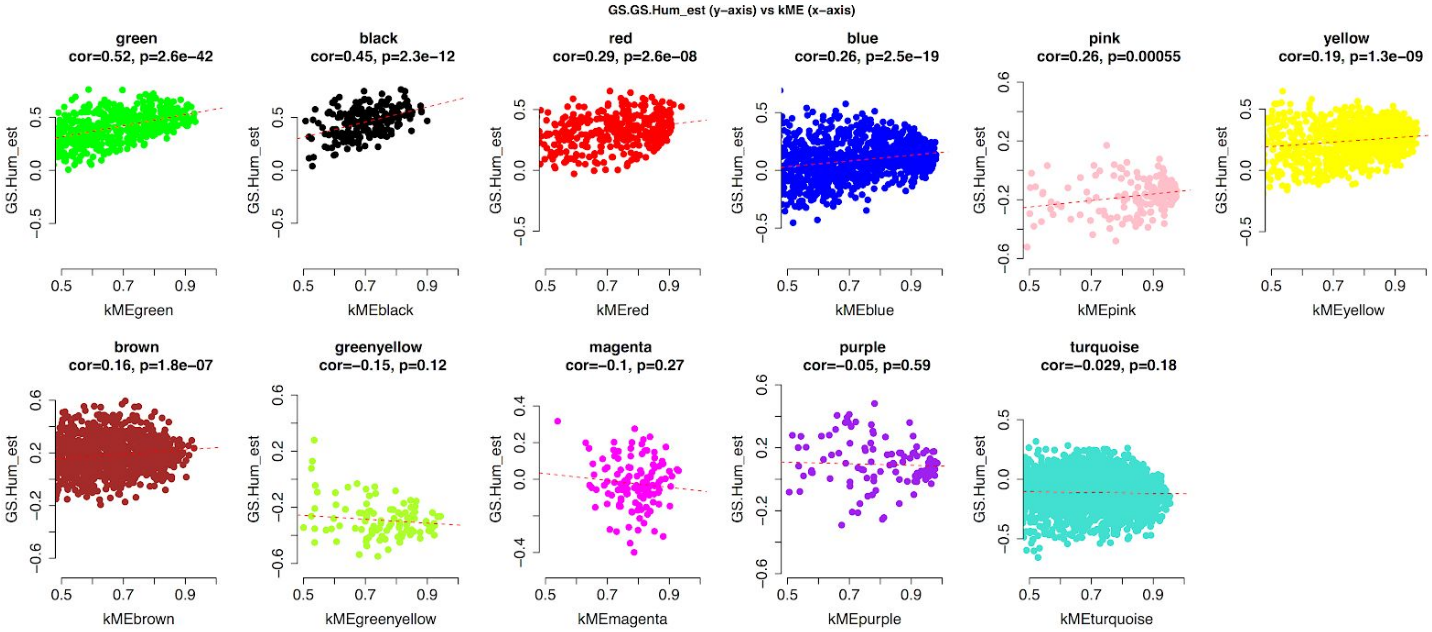

Supplementary Figure 5.

Coexpression centrality and Expression correlation with colony behavioral variation.

For the 11 coexpression modules, the coexpression centrality of a gene (kME, x-axis) vs. how correlated its expression was with colony sensitivity to humidity (GS.Hum_est, y-axis). Pearson correlation coefficients and p-values are shown. The trend (in all 7/11 modules with a regression p-value < 0.05) is that loci with higher module centrality have a higher correlation with colony sensitivity of foraging activity to humidity.

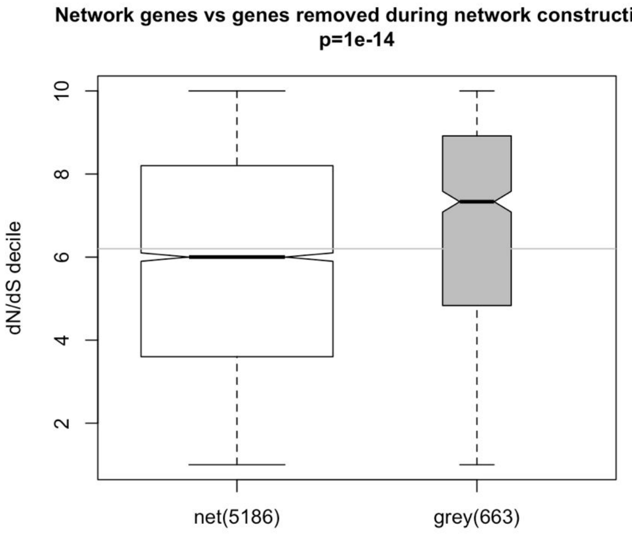

Supplementary Figure 6.

Bar chart reflecting the dN/dS decile composition (y-axis) for loci included in coexpression networks (white bar) vs. excluded from coexpression networks (gray bar). Loci with no identifiable homologs were not included in this comparison. Loci included in coexpression networks have significantly lower dN/dS than loci excluded from networks during coexpression module fitting.

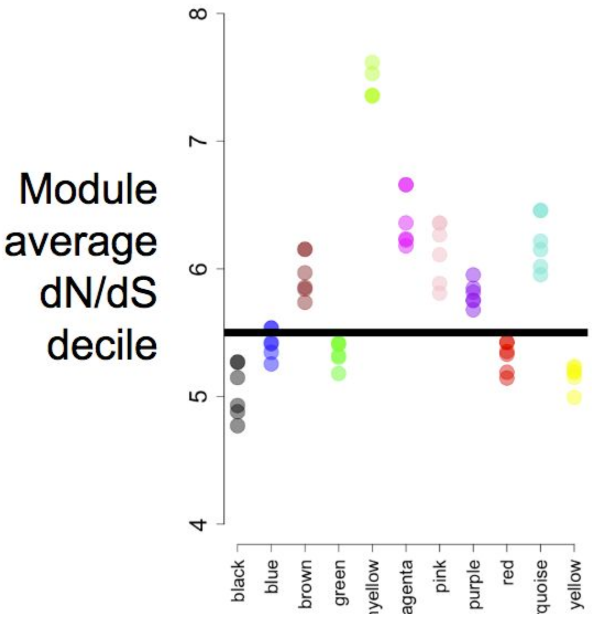

Supplementary Figure 7.

Scatterplot of average dN/dS decile by coexpression module. Y-axis is the average dN/dS decile of a module. Each of the six points per module are the values calculated separately for each of the six species used for evolutionary comparisons (Methods) . The black line is at the expected value of 5.5.

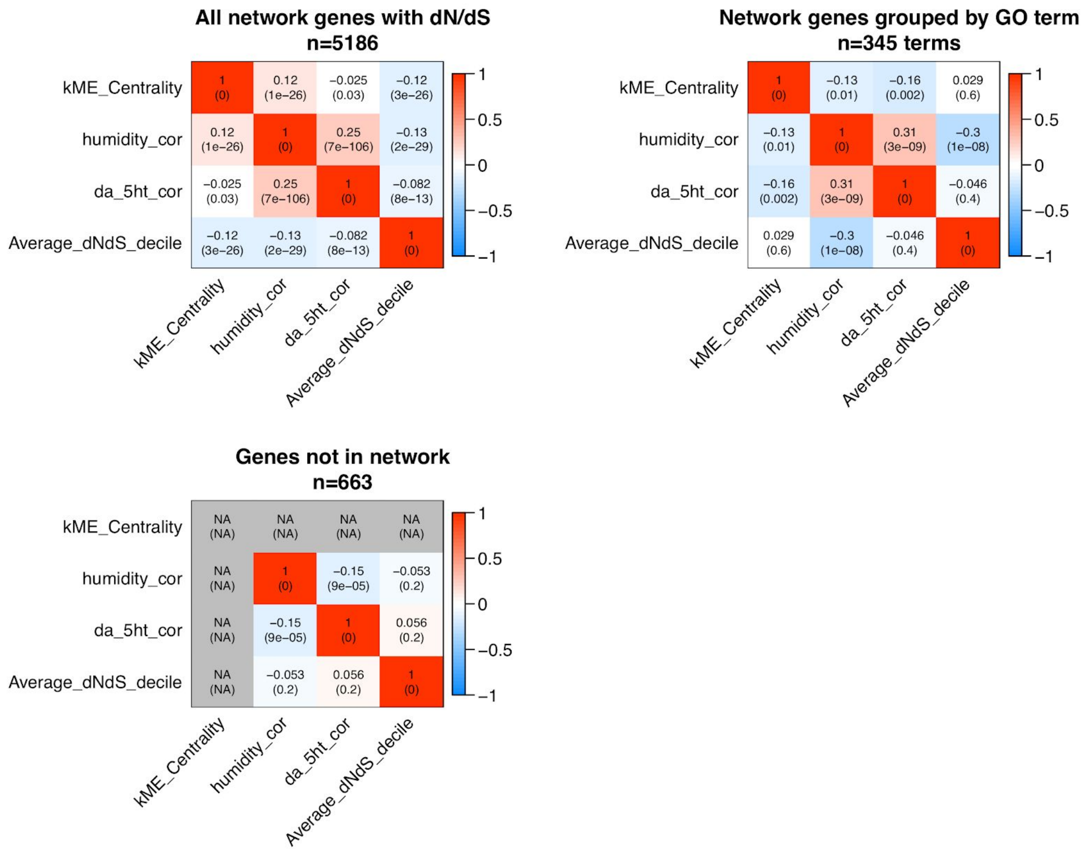

Supplementary Figure 8.

Heatmap of correlations among expression patterns, coexpression centrality, and degrees of evolutionary coding sequence constraint. Heatmap cells show Spearman correlations among module centrality (kME; defined as the correlation between a gene's expression profile and the first principal component of its module, aka the module eigengene, or ME), correlation to sensitivity of foraging activity to humidity (humidity_cor), dopamine-to-serotonin ratio (da_5ht_cor), and dN/dS decile (Average_dNdS_decile). Darker shades of red and blue correspond to more positive and negative correlations, respectively, and white corresponds to zero correlation. Top left: All genes in the final coexpression network with defined dN/dS ratios; Top right: Average values of genes within GO terms that were enriched in modules, i.e. numbers in this plot represent correlations between the last 4 columns of Table A; Bottom left: Genes that were pruned from the network during optimization for densely interconnected modules, as such they do not have kME values since they were not assigned to a module.

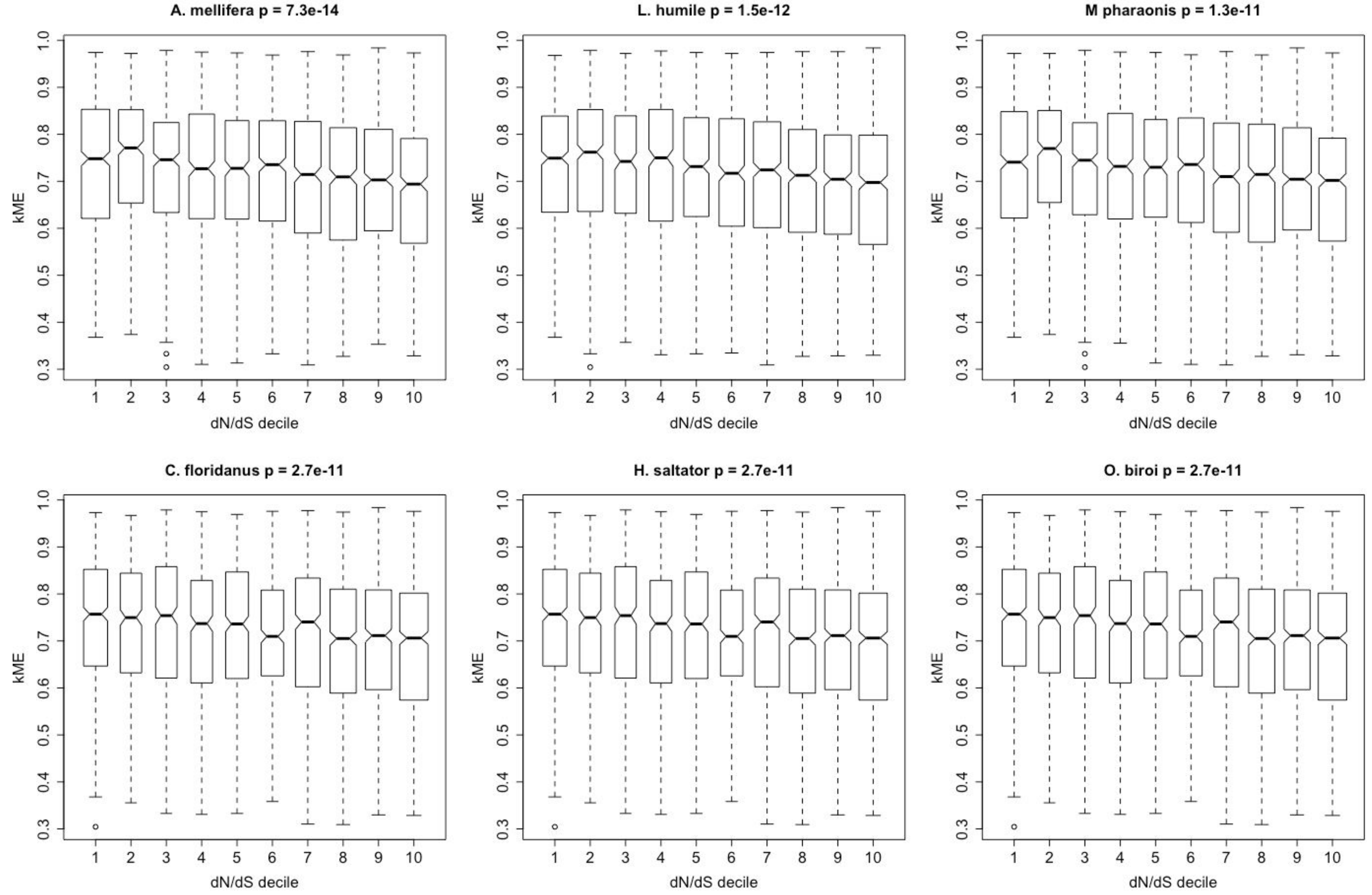

Supplementary Figure 9.

Barplot of modules coexpression centrality versus degree of coding sequence constraint. Relationships are shown separately for each of the six species involved in dN/dS calculations. Module centrality (kME) is on the Y-axis, and distributions are plotted as a function of locus dN/dS decile in that species (X-axis). P-values next to species name show regression statistics, all regressions show that lower dN/dS loci are significantly more central to coexpression loci.

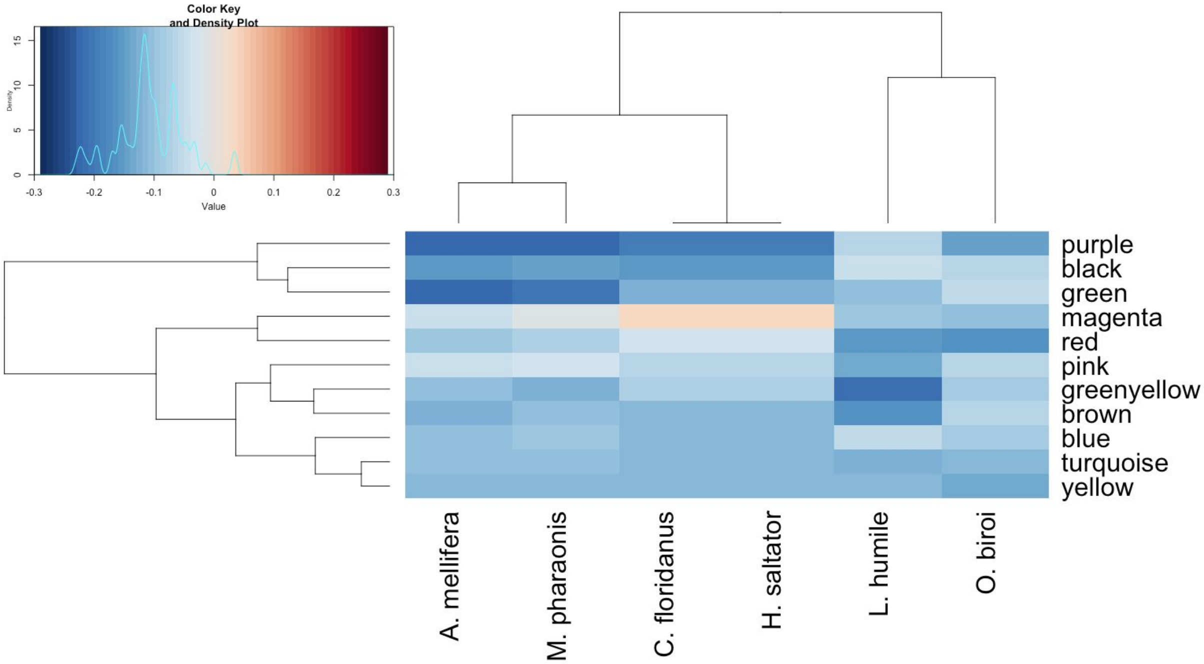

Supplementary Figure 10. Heat map showing for each species,the average correlation coefficient between locus centrality and dN/dS. Correlation between coexpression centrality and dN/dS range from negative (blue) to positive (orange). For almost all modules when compared separately to most species, there is a negative relationship between coexpression centrality and dN/dS, meaning that there is a positive association between centrality in a coexpression module and degree of sequence conservation over evolutionary time.

Supplementary Tables

Supplementary Table 1: Colony traits.

Colony	Sensitivity to Humidity	DA:5HT ratio
D19	142.6475908	3.977
D24	31.48433296	4.046
D25	44.67682713	3.867
D26	265.0094372	3.902
D27	153.5498529	4.029
D29	40.13467226	4.257
D30	26.92988938	3.802
D33	205.3109578	3.849
D36	99.47928684	4.026

For the 9 colonies, the trait values are given. “Sensitivity to humidity” is given in units of “number fewer foraging trips taken per degree decrease in relative humidity”. Dopamine to Serotonin ratio is from HPLC data. Both traits were calculated in Friedman et al. 2018, *iScience.*

Supplementary Table 2: Coexpression module summary.

Color	# Loci	Average dN/dS decile	Different than 5.5? P =	Correlation with Sensitivity of foraging activity to humidity	Different than 0? P; corr ≠ 0	Correlation with average DA:5HT	Different than 0? P; corr ≠ 0
black	220	5.26	0.28	**0.2813**	**< 0.0001**	**0.1561**	**< 0.0001**
blue	1157	5.54	0.74	**0.1057**	**< 0.0001**	**-0.0074**	0.0854
brown	1053	6.15	**1.71E-09**	**0.1383**	**< 0.0001**	**0.08711**	**< 0.0001**
green	592	5.42	0.56	**0.2618**	**< 0.0001**	**0.05927**	**< 0.0001**
greenyellow	108	7.36	**9.24E-09**	**-0.1496**	**< 0.0001**	**-0.081**	**< 0.0001**
magenta	123	6.66	**5.52E-05**	**0.0473**	**0.0043**	**0.0972**	**< 0.0001**
pink	173	6.35	**0.0017**	**-0.0584**	**0.0008**	**-0.03**	**0.0027**
purple	120	5.75	0.45	**0.0984**	**< 0.0001**	**0.0355**	**0.0006**
red	355	5.42	0.66	**0.2303**	**< 0.0001**	**0.02**	**0.012**
turquoise	2182	6.46	**2.20E-16**	**-0.02725**	**< 0.0001**	**0.012**	**< 0.0001**
yellow	1002	5.19	**0.0045**	**0.1783**	**< 0.0001**	**-0.0159**	**0.0004**

Coexpression modules are named by color, and the number of loci in each module is given. For degree of coding constraint, the dN/dS decile average is given. Teal is significantly lower dN/dS than expected, Yellow is significantly higher dN/dS than expected (both test $p<0.05$, one-sample t-test for inequality with 5.5, decile average of N=6 comparative species). The column “Correlation with Sensitivity of foraging activity to humidity” is the average correlation of the expression of the loci in that module with colony behavioral traits. All values are significantly different than zero (one-sample t-test), and modules with average correlations of magnitude ≥ 0.1 are colored Blue for positive correlation, and Red for negative correlation. The column “Correlation with average DA:5HT” is calculated and colored as with the previous behavior trait, but here for colony average forager brain dopamine to serotonin ratio.
